# Impact of biological maturation on physical tests performance in Portuguese elite male academy soccer players

**DOI:** 10.5114/biolsport.2026.161099

**Published:** 2026-07-06

**Authors:** Nuno Ribeiro, Oliver Gonzalo-Skok, Joao Valente dos Santos, Joao R. Pereira, Mauro Mandorino, Jo Clubb, Marco Beato, Jamie Salter, Hadi Nobari, Nuno Loureiro, Ruben Ferreira, Joao Pedro Araujo, Francisco Tavares

**Affiliations:** 1 Faculty of Physical Education and Sport, Lusofona University, 1749-024 Lisbon, Portugal; 2 CIDEFES, Research Center in Sports, Physical Education, Exercise and Health, Universidade Lusofona, 1749-024 Lisbon, Portugal; 3 Medical and Performance Department, Sporting Clube de Portugal, Lisbon, Portugal; 4 Department of Communication and Education, Universidad Loyola Andalucia, Seville, Spain; 5 CIFI2D, Centre of Research, Education, Innovation and Intervention in Sport, University of Porto, Porto, Portugal; 6 COD, Center of Sports Optimization, Sporting Clube de Portugal, 1600-464 Lisbon, Portugal; 7 Performance and Analytics Department, Parma Calcio 1913, 43121 Parma, Italy; 8 Department of Movement, Human and Health Sciences, University of Rome Foro Italico, Piazza L. de Bosis 6, 00135 Rome, Italy; 9 Global Performance Insights Ltd, London, United Kingdom; 10 School of Health and Sports Sciences, University of Suffolk, Ipswich IP4 1QJ, United Kingdom; 11 School of Science, Technology and Health, York St John University, York, United Kingdom; 12 LFE Research Group, Department of Health and Human Performance, Faculty of Physical Activity and Sport Science (INEF), Universidad Politécnica de Madrid, C/Martín Fierro 7, 28040, Madrid, Spain

**Keywords:** Maturation, Physical performance tests, Performance indicators, Field testing, Young athletes, Long-term athletic development

## Abstract

This study examined the influence of biological maturation on the physical performance of highly skilled U-14 – U-17 male soccer players, specifically focusing on sprinting, jumping, and aerobic capacity. Sixty-seven players from a professional academy in Portugal participated in two seasons (2021–2023). Maturation was assessed at six time points, and players were categorized into pre- and circa-peak height velocity (PHV) and post-PHV groups. Owing to the small pre-PHV sample size (n = 4), these were combined with the circa-PHV group for the analysis. Key physical attributes were measured, and maturity was assessed using the predicted percentage of adult height. Mixed-effects models indicated that chronological age significantly influenced physical performance, with older players demonstrating a superior change in direction speed (p < 0.001) and sprint performance (p < 0.001), whereas maturity status had no additional effect. Maturity status interacted with age in the jumping height test (p = 0.001), with post-PHV players showing greater improvement. Chronological age and biological maturity significantly influenced endurance (p = 0.001) and maximal strength (p = 0.020), with post-PHV players exhibiting greater gains. Chronological age and biological maturation significantly affected the physical performance of young soccer players. Relying solely on age may overlook developmental factors and underscore the need for tailored training and assessment strategies.

## INTRODUCTION

Soccer academies play a critical role in identifying and developing young talent with the potential to compete at senior levels and secure professional contracts [1]. These academies provide essential resources, training, and guidance to help clubs cultivate promising players [2]. A key phase in this process is the selection and rejection of players, which frequently occurs between the ages of 14 and 15 [3]. At these ages, substantial variations in players’ maturity levels can influence talent selection decisions [4–6]. Research has shown that elite youth soccer academies observe differences of five to six years between the biological and chronological ages of players [7]. Such disparities in biological maturation can lead to substantial variations in size and performance [8]. These differences in maturity status profoundly affect the physical qualities crucial for soccer performance, including strength, power, speed, agility, and endurance [4–6, 9]. Therefore, an accurate assessment of players’ biological age is paramount, given the potential discrepancies between their chronological and biological ages [10, 11]. Understanding how biological maturation influences physical performance is essential for practitioners working in youth soccer, as it may affect talent identification processes, training prescription, and long-term player development.

Physical quality refers to an individual’s ability to possess or develop skills that allow them to participate in physical activities [12]. Assessments of fitness components, such as strength, power, endurance, and speed, are considered measures of physical quality [13]. The physical qualities most frequently examined in soccer are cardiorespiratory fitness, muscular power, maximal strength, movement speed, agility, balance, and body composition [14]. While evaluating these qualities is essential, it is equally important to understand their relationship with maturation status [15], as this can influence talent identification and performance optimization [1]. The interaction between biological maturation and physical performance is complex, as developmental changes in neuromuscular, hormonal, and morphological characteristics may influence the progression of physical qualities during adolescence. Biological maturation, distinct from simple growth, is a complex and ongoing process that begins at conception and culminates in adulthood [3, 6]. It varies widely among individuals in terms of timing, tempo, and sequence of developmental events. Maturity status represents an individual’s level of maturation at a given time, whereas the timing and tempo of maturation refer to the occurrence and rate of specific developmental milestones, respectively [11]. Consequently, consistent monitoring and evaluation of growth and maturation are critical for understanding their effects on player development.

However, evaluating biological maturation is challenging because of the need for specific indicators for each biological system and different definitions of maturation across systems [16]. Skeletal maturation is widely regarded as the most accurate and reliable measure of biological maturation and aids in understanding the developmental trajectories of players [10]. Soccer academies often use somatic equations derived from anthropometric measurements to estimate age at peak height velocity (PHV) and predict adult height [17]. Despite the utility of these methods, there are concerns about discrepancies between predicted and actual chronological age at PHV, particularly for boys far from PHV [18]. The Khamis–Roche method, which utilizes reference data from the Fels Longitudinal Study [19], is used to forecast adult height and categorize youth into bands based on the percentage of predicted adult height (PPAH), such as ≥ 85.0% and < 90.0% or ≥ 90.0% and < 95.0% [11, 20, 21]. These bands are intended to capture the adolescent growth spurt but can be adjusted as needed, with adult height reference values set for age 18 [11, 20]. Despite some expected errors (approximately 2%) in anthropometric measurements, the Khamis–Roche method has shown promise for evaluating somatic maturation, aligning with the error values reported in previous studies [16, 21].

Longitudinal studies that track the evolution of physical qualities over two or more athletic seasons are particularly valuable from a long-term athlete development (LTAD) perspective. These studies allowed for the observation of developmental progression by consistently monitoring the same group of athletes (pre-PHV/PHV vs. post-PHV) throughout the study period. Although the effects of biological maturation on the physical performance of young soccer players have been extensively studied, much of the current literature relies predominantly on cross-sectional research designs. Consequently, there is limited information on how biological maturation interacts with chronological age to influence the longitudinal development of multiple physical qualities across competitive seasons within elite academy environments. Understanding these developmental patterns may help practitioners interpret performance changes during adolescence and support informed decision making in elite youth soccer academies.

Therefore, this study aimed to investigate the influence of biological maturation on the physical performance of elite young male soccer players. Specifically, this study conducted a longitudinal examination of physical qualities across two consecutive soccer seasons, focusing on U14 and U17 players in a high-level soccer academy. We hypothesized that (1) chronological age would be associated with improvements in sprint and change of direction (COD) performance, (2) biological maturation would interact with chronological age to explain strength and power performance (e.g., countermovement jump and isometric mid-thigh pull), and (3) more biologically mature players would demonstrate superior endurance performance. The secondary objective was to establish reference values for several physical performance tests in academy soccer players. These findings may assist practitioners in identifying the physical performance qualities most influenced by biological maturation during adolescence.

## MATERIALS AND METHODS

### Experimental Approach to the Problem

This study used a longitudinal approach with repeated measurements. Sixty-seven young male soccer players from a professional soccer academy in Portugal were enrolled to investigate aspects of development, maturation, and physical qualities, including muscular strength, COD, linear speed, and aerobic capacity. The assessment of biological maturity and physical qualities began during the initial evaluation of the 2021–2022 season in the first week of training and continued until the first week of May 2023 (Table 1). The study involved six assessment points across the 2021–2022 and 2022–2023 sports seasons, with three evaluations per season, three months apart (Table 1).

**TABLE 1:** Assessment during the two sports seasons.

Season	2021–22			2022–23		
**Year**	**2021**	**2022**	**2022**	**2022**	**2023**	**2023**
**Month**	August	January	May	August	January	May
**Week**	1^st^	1^st^	1^st^	1^st^	1^st^	1^st^
**Assessment Number**	1^st^	2^nd^	3^rd^	4^th^	5^th^	6^th^

Players were categorized into two groups based on their biological maturation stage using the Khamis–Roche method [21]: Group 1 comprised athletes in the pre- and circa-PHV periods (< 95.0% PAH), while Group 2 consisted of athletes in the post-PHV period (≥ 95.0% PAH). Owing to the limited number of players in the pre-PHV stage (n = 4), pre-PHV and circa-PHV players were combined into a single group (pre- and circa-PHV) to avoid statistical instability and insufficient statistical power for comparisons between the maturation groups. This distribution likely reflects the typical maturation distribution observed in elite youth soccer academies.

### Subjects

Sixty-seven male soccer players born between 2002 and 2007 at a professional soccer academy in Portugal were assessed. The players had an average age of 15.1 years (± 1.1 years), ranging from 12.0 to 18.1 years, a mean height of 174.9 cm (± 8.0 cm), and an average body mass of 64.1 kg (± 8.5 kg) (Table 2). The participants were from the U14, U15, U16, and U17 age groups in their first assessment during the 2020–21 season. All players participated without dropouts or missing data during the assessment. The inclusion criteria were as follows: (1) at least three years of soccer experience, (2) registered in the academy/club for at least two seasons, and (3) did not use supplements that could affect growth and maturation. The exclusion criteria were as follows: (1) players injured during the off-season and (2) players who did not participate in the study. The players had training/match experience of 5.5 ± 1.1 years at the national and international levels. This study was approved by the Ethics Committee of the Faculty of Physical Education and Sports, University of Lusófona. The purpose, procedures, requirements, benefits, and hazards of the study were communicated to the parents/custodians who provided written informed consent. This study adhered to the ethical standards of the Declaration of Helsinki for human research.

**TABLE 2:** Anthropometric, Body Composition and Maturity characteristics from under-14 to under-17.

Assessment	N	Chronological Age (y)	Height (cm)	Leg Length (cm)	Body Mass (kg)	Body Mass Index (BMI)	Σ 8 skinfolds (mm)	% Predicted Adult Height (%)
1	67	14.3 ± 0.93	172.0 ± 8.53	84.0 ± 4.28	59.6 ± 8.88	20.0 ± 1.73	49.9 ± 9.61	94.7 ± 3.17
2	67	14.7 ± 0.91	173.6 ± 8.08	84.5 ± 4.18	62.1 ± 8.57	20.5 ± 1.70	51.0 ± 8.53	95.9 ± 2.81
3	67	14.9 ± 0.94	174.4 ± 7.93	84.8 ± 4.34	63.1 ± 8.26	20.7 ± 1.73	51.9 ± 8.64	96.5 ± 2.68
4	67	15.3 ± 0.93	175.9 ± 7.58	85.2 ± 4.19	64.9 ± 7.86	20.9 ± 1.65	51.1 ± 7.25	97.3 ± 2.32
5	67	15.6 ± 0.93	176.4 ± 7.42	85.5 ± 4.19	66.7 ± 7.43	21.4 ± 1.56	51.2 ± 7.76	98.0 ± 2.02
6	67	15.9 ± 0.91	177.1 ± 7.32	85.7 ± 4.29	67.9 ± 7.29	21.6 ± 1.51	53.9 ± 10.10	98.5 ± 1.81

### Procedures

The following sequence was used throughout the assessments: (1) anthropometry and body composition; (2) countermovement jump (CMJ); (3) isometric mid-thigh pull (IMTP); (4) change of direction ability (“L-test”), (5) sprint test (30 m), and (6) aerobic fitness test, 1200 m shuttle run test (The Bronco test). All athletes were familiar with all physical tests. A comprehensive overview of these examinations was provided. Controlling for learning effects is crucial, particularly in assessments that involve complex technical requirements. The tests were conducted over a week on different days, with 24 h between the physical performance assessments. This sequence progressed from least to most physically demanding to minimize the potential impact of fatigue on subsequent assessments. Before each test, a general and specific warm-up was performed, involving movements similar to those in the test, to increase reliability [22]. For the general warm-up, the players completed 10 min of light jogging, 5 min of dynamic stretching including lunges, divet stretching, and lateral squats, and 5 min of moderate-to-high-intensity movements, such as high knees, butt kicks, cariocas, accelerations, decelerations, sprints, and directional changes. For the Bronco test, players completed a standardized warm-up of 3 min of submaximal jogging combined with dynamic stretching and one practice lap at approximately 50% maximal effort [23]. For the remaining tests, the players performed two warm-up repetitions specific to the objective of each test. For tests (2), (3), (4), (5), and (6), three attempts were made to obtain the best results. A 3-min rest interval was provided between attempts. During all tests, players received consistent verbal encouragement to produce maximal effort [24] from strength and conditioning coaches.

#### Anthropometry and Body Composition

Anthropometric evaluations were performed following the standardized guidelines set by the International Society for the Advancement of Kinanthropometry [25]. A certified ISAK Level I technician experienced in these procedures conducted all assessments to ensure compliance with standardized methods. Measurements were taken in the morning under controlled conditions, after an overnight fast, and at least 24 h without training, to reduce immediate physiological effects. The anthropometric parameters measured included body weight, standing and sitting heights, and eight skinfold thicknesses (triceps, subscapular, biceps, suprailiac, abdominal, supraspinal, thigh, and calf). An anthropometer (Seca model 206; Seca, Hamburg, Germany) with a precision of 0.1 cm was used to measure standing and sitting heights. Body weight was determined using a calibrated electronic scale (Tanita model BC-601; Tanita Corporation, Tokyo, Japan) with a precision of 0.1 kg. Body mass index (BMI) was calculated by dividing body weight by the square of height (kg/m^2^). Skinfold thicknesses were measured with a Harpenden skinfold caliper (British Indicators Ltd., London, UK) with an accuracy of 0.1 mm. Each anthropometric measurement was performed twice, and the average of the two was used for further analysis. The intraobserver technical error of measurement (TEM) was maintained within acceptable ISAK limits (< 1% for height and body weight; < 5% for skinfold thickness). If the difference between repeated measurements exceeded these limits, a third measurement was taken, and the median value was used. The use of standardized ISAK procedures, duplicate measurements, and predefined error thresholds ensured high intra-observer reliability and consistency in measurements.

#### Microcycle Structure

Players aged 14–17 years had a weekly training schedule during the competitive season that included one match designed by a match day (MD), followed by a day off (MD+1/-6), and a regeneration session (MD+2/-5). The training sessions leading up to the match were structured as follows: MD+3/-4, emphasized through positional drills and small-sided games. MD+4/-3 concentrated on tactical preparation for the upcoming match, featuring moderate-intensity positional exercises. MD+5/-2 incorporated activation drills that mimicked certain technical and tactical aspects and concluded with practice on set pieces. MD+6/-1 was the day off for all squads. Training sessions included physical, technical, and tactical components. The microcycle or weekly training schedule may be modified based on developments on the day of the match. Young soccer players participated in four 90-min training sessions weekly, with matches typically held on weekends. Matches for U14–U15 lasted 80 min, while those for U16–U17 lasted 90 min and were conducted on outdoor artificial turf fields with 11 players per side. The players in the academy participated in an average of 12 h of combined training per week, which included four to five sessions of soccer, strength and power exercises (one to two sessions), and speed and COD training (one to two sessions), along with one competitive match per week.

#### Playing Position

In total, 67 young male players were categorized as goalkeepers (n = 7), central center-backs (n = 11), winger-backs (n = 10), central midfielders (n = 19), wingers (n = 13), and forwards (n = 7). Although it is acknowledged that young soccer players aged 12–18 years may adopt various positions [26], the criterion for defining the position of each player was the highest number of sessions in that position.

#### Biological Maturation

The Khamis–Roche method was used to define the biological maturation of each player (pre-, circa-, and post-PHV) [21]. The percentage of observed adult height was used to indicate maturity status [3, 6]. Height, body mass, chronological age, and average parent height were used to predict the adult height of each player and ascertain their biological maturity [21]. The heights of the biological parents of each player were self-reported and adjusted for overestimation using previously published equations [27]. Using the Khamis–Roche method, it is possible to predict the final adult height of men between 4.0 and 17.5 years old, considering a median error associated with the use of the 2.2 cm method, that is, an average error that can vary between 0.8 and 2.8 cm [21]. This method represents a practical and noninvasive alternative for longitudinal monitoring in applied elite settings. As indicated earlier, players were divided into three groups according to their maturity status using the percentage of their PPAH. Thus, circa-PHVs accounted for 88–95% of the expected adult stature. Subsequently, the pre-PHV was < 88% and post-PHV was > 95% [11, 28]. The two groups were considered for data analysis based on biological maturation (maturity status). Group 1 comprised young soccer players with a predicted adult height percentage of up to 95% (< 95.0% PAH), and Group 2 comprised players with a predicted adult height percentage of ≥ 95% (≥ 95.0% PAH). The composition of each group was adjusted throughout the assessment period. Regarding the sample distribution between the two groups (pre-PHV/PHV and post-PHV), at the first evaluation, Group 1 consisted of 32 players and Group 2 consisted of 35 players.

#### Countermovement Jump Test

The CMJ test was performed after the IMTP test. The participants performed countermovement jumps with their hands placed on their hips while maintaining an upright trunk position. Participants were allowed to freely select their countermovement depth and speed and were instructed to jump as high as possible [30]. Each player completed three jumps with 1 min of rest between attempts [29]. Jump height was measured using a contact platform (Chronopic^®^, Chronojump Boscosystem, Barcelona, Spain), and the associated software was used to calculate jump height [30]. The highest jump height (cm) recorded across the three trials was used for the analysis. The CMJ protocol has been shown to be a valid and reliable measure of neuromuscular performance in young athletes, with high test–retest reliability (ICC = 0.83) [31].

#### IMTP Test

The IMTP test was performed with the athlete standing on a 1000 Hz force platform (Pasco, Rosedale, USA; data analyzed using NMP ForceDecks, London, UK) with a fixed bar positioned at mid-thigh height. The players adopted a posture similar to the second pull of the clean, with the trunk upright, shoulders positioned above or slightly behind the bar, and feet placed under the bar. Knee and hip joint angles were standardized at approximately 125–145° and 140–150°, respectively [34]. The players completed a standardized warm-up consisting of three progressive efforts (50%, 75%, and 90% of maximal effort), each lasting 3 s with 60 s of rest between efforts [32]. Subsequently, three maximal IMTP trials were performed, each lasting 3 s with 60 s of rest between trials. Players were instructed to pull as hard as possible after a verbal countdown (“3, 2, 1, pull!”). Peak force was defined as the highest force value recorded during the 3-s trial. The best value from the three trials was retained for analysis. Relative force was calculated by dividing the net peak force (peak force − body mass) by the player’s body mass. The IMTP test has been shown to be a reliable method for assessing maximal isometric strength, demonstrating high test–retest reliability (ICC > 0.90) in athletic populations [32].

#### Change of Direction Ability

The “L-test” assessment was conducted on an indoor synthetic surface following an established protocol to measure speed and directional change ability. This protocol adhered to the original method [33] and was used in subsequent studies [34]. The test consisted of an “L-shaped running course formed by three cones placed 5 m apart. Players sprinted from the start line to the first cone, changed direction toward the second cone, and then returned to the start line, running around the cones to complete the “L-shaped path. Electronic timing gates (Witty Gate, Microgate, Italy) were used to record performance, with the photoelectric cell positioned approximately 1 m above the ground. Participants started from a standing “athletic position” with the front foot placed approximately 0.30 m behind the first timing gate. Each player performed three trials with 2 min of rest between attempts. The fastest time recorded to the nearest 0.01 s was retained for analysis. COD tests using electronic timing systems have previously demonstrated good reliability in athletic populations [35].

#### Sprint Test

Sprint performance was assessed over 30 m using electronic timing gates (Witty Gate, Microgate, Italy). The test was conducted on an indoor synthetic surface with timing gates positioned at the start line (0 m) and 30 m. The photoelectric cell was positioned 1.5 m from the receiver. The players started from a standing position with the front foot placed approximately 0.30 m behind the first timing gate and without any countermovement. Each player performed three maximal sprint trials with 4 min of passive recovery between attempts [36]. Strong verbal encouragement was provided during each trial. The fastest sprint time (0–30 m) recorded to the nearest 0.01 s was retained for analysis. Linear sprint testing using electronic timing gates has demonstrated excellent reliability in young soccer players (ICC > 0.90) [37].

#### Maximal Aerobic Speed Test

Maximal aerobic speed (MAS) was assessed using a 1200 m shuttle run test (commonly referred to as the Bronco test). The participants started at the start line and performed repeated shuttle runs of 20, 40, and 60 m (i.e., 20 m and back, 40 m and back, and 60 m and back), completing five consecutive sequences for a total distance of 1200 m [38]. The participants were required to fully cross or touch each line during the test. All players were familiarized with the protocol prior to testing to ensure proper pacing and execution [38]. The total time required to complete each test was recorded. The MAS was calculated by dividing the total distance by the recorded time, with a correction factor applied using the equation MAS = 1200 / (time in seconds – 20.3) [39]. The 1200 m shuttle run (Bronco test) has previously demonstrated good reliability as a field-based measure of high-intensity running performance in team-sport athletes (ICC > 0.80) [40].

### Statistical Analysis

Initial zero-order Pearson correlations were used to inspect the potential overlap between the performance metrics. The correlations ranged from 0.19 to 0.61, indicating moderate relationships, suggesting a degree of shared variance while preserving sufficient independence among variables. These results also indicate the absence of problematic multicollinearity, supporting the inclusion of all performance metrics in subsequent analyses. Descriptive statistics for all performance measures are reported as mean ± standard deviation. To examine changes in performance metric power [CMJ test], maximal strength [IMTP test], change of direction (COD) [L-test], sprint ability [30-m test], and maximal aerobic speed [Bronco test] as a function of chronological age and maturity status (pre- and circa-PHV players were coded as 0, and post-PHV players were coded as 1), a two-level growth model was employed. At Level 1, each athlete’s successive measurements over time were modeled to capture individual changes at each time point, accounting for random errors. These individual changes were expressed as centered values (grand-mean centering) to improve interpretability and reduce multicollinearity. At Level 2, differences in growth trajectories between groups defined by maturity status were examined. To make inferences regarding the true (population) values of the effects of age and maturity status on physical performance, the 95% confidence interval (CI) for each effect was also calculated.

Mixed-effects linear models were used to investigate the effects of chronological age and maturity status on performance. We constructed three models for each dependent variable: (1) chronological age as the only fixed effect; (2) age and maturity status as fixed effects; and (3) age, maturity status, and their interaction as fixed effects. Player ID was included as a random effect in all models to account for repeated measures within subjects. The residuals from each model were assessed for normality using histograms and Q–Q plots, and homoscedasticity was evaluated using residual plots. Multicollinearity among predictors was checked using Variance Inflation Factors (VIFs). Model fit was compared using log-likelihood values and Akaike Information Criterion (AIC). The model with the lowest AIC was considered the best-fitting model. The magnitude and direction of the associations were interpreted using the fixed-effect estimates (B coefficients) obtained from the mixed-effects models, reported together with their standard errors and 95% confidence intervals. Statistical analyses were performed using the Statistical Package for the Social Sciences (version 28.0; SPSS Inc., Chicago, IL, USA). The threshold for statistical significance was set at *p* < 0.05.

## RESULTS

The correlations ranged from 0.19 to 0.61, indicating moderate relationships among the performance variables and suggesting that each test retained some level of independent properties relevant to short-term performance. For the CMJ test, both chronological age and maturity status significantly influenced performance, with a significant interaction between these variables (p = 0.001) (Figure 1). Post-PHV players showed greater improvements in jumping height with age than their pre- and circa-PHV counterparts. Similarly, maximal strength assessed using the IMTP test showed significant effects of age, maturity status, and their interaction (p = 0.020) (Figure 2). The interaction indicated that strength gains were more pronounced in post-PHV players, suggesting that biological maturation enhanced the rate of strength development beyond the effect of age alone. Chronological age emerged as a significant predictor of COD speed, as measured by the L-test, with older players generally demonstrating better performance (p < 0.001) Figure 3). The inclusion of maturity status and its interaction with age did not significantly improve the model (both p > 0.05). A similar pattern was observed for the 30-m sprint test, in which chronological age was the main determinant of performance, with older players running faster (p < 0.001) (Figure 4). Maturity status and its interaction with age did not significantly contribute to the predictive model (both p > 0.05). Endurance performance, assessed using the Bronco test, was significantly influenced by both age and maturity status, with a significant interaction between these variables (p = 0.001) (Figure 5). Post-PHV players demonstrated greater improvements in endurance over time.

**FIG. 1:**
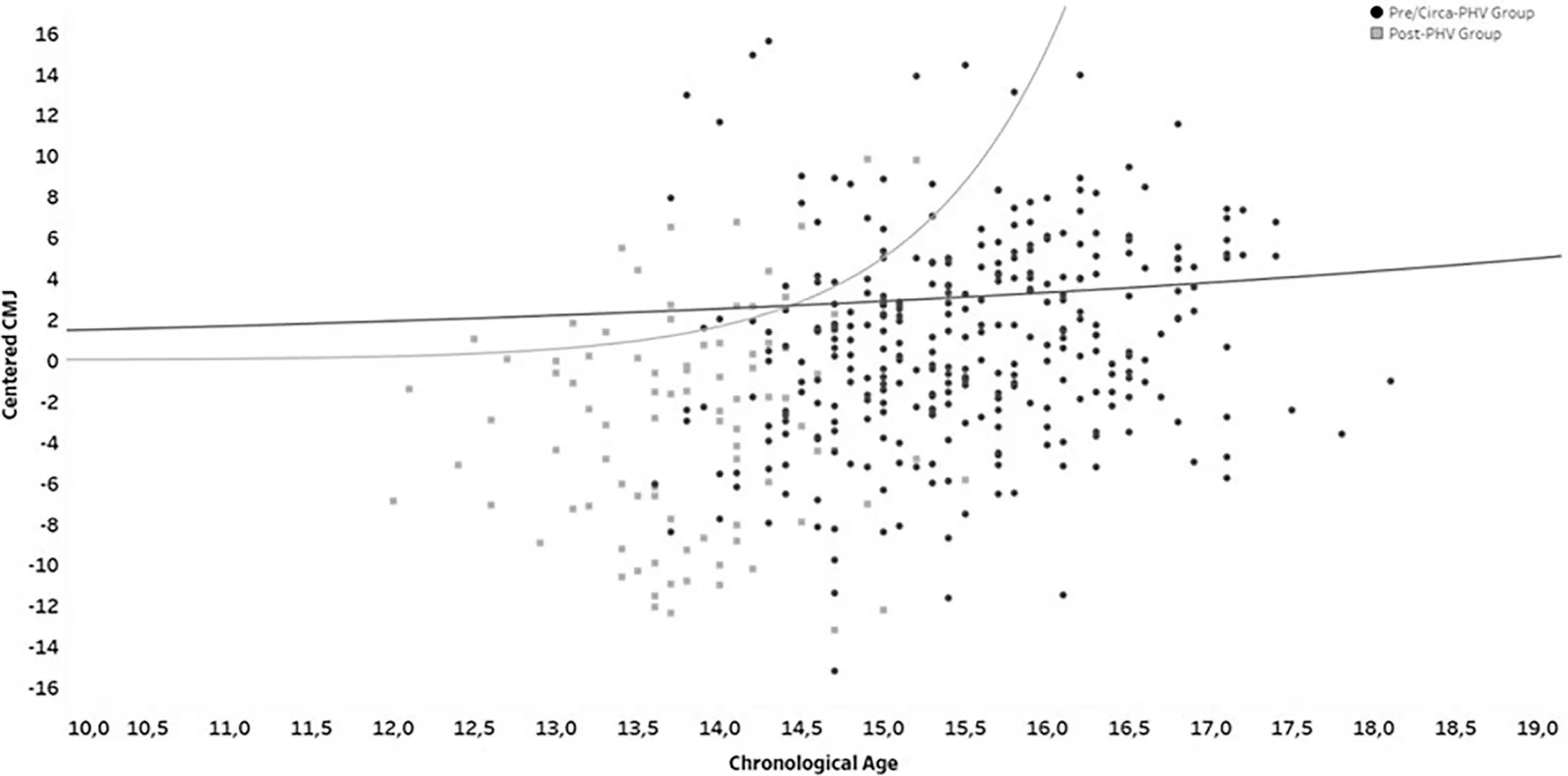
Relationship between chronological age and centered countermovement jump (CMJ) performance according to biological maturity status (pre/circa-PHV vs post-PHV). **Key.** CMJ = countermovement jump; PHV = peak height velocity; pre-PHV = pre-peak height velocity; circa-PHV = circa-peak height velocity; post-PHV = post-peak height velocity.

**FIG. 2:**
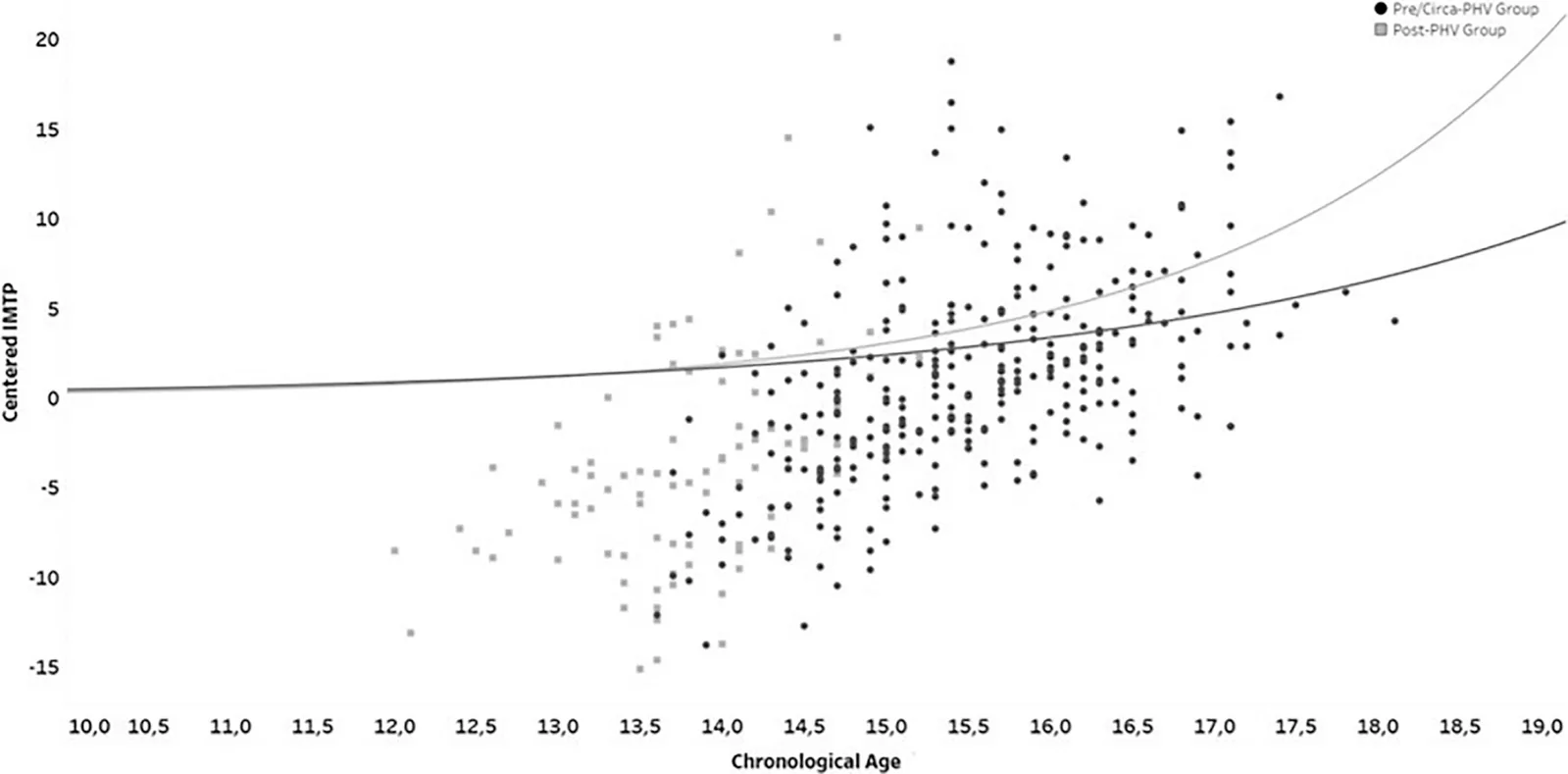
Relationship between chronological age and centered isometric mid-thigh pull (IMTP) performance according to biological maturity status (pre/circa-PHV vs post-PHV). **Key.** IMTP = isometric mid-thigh pull; PHV = peak height velocity; pre-PHV = pre-peak height velocity; circa-PHV = circa-peak height velocity; post-PHV = post-peak height velocity.

**FIG. 3:**
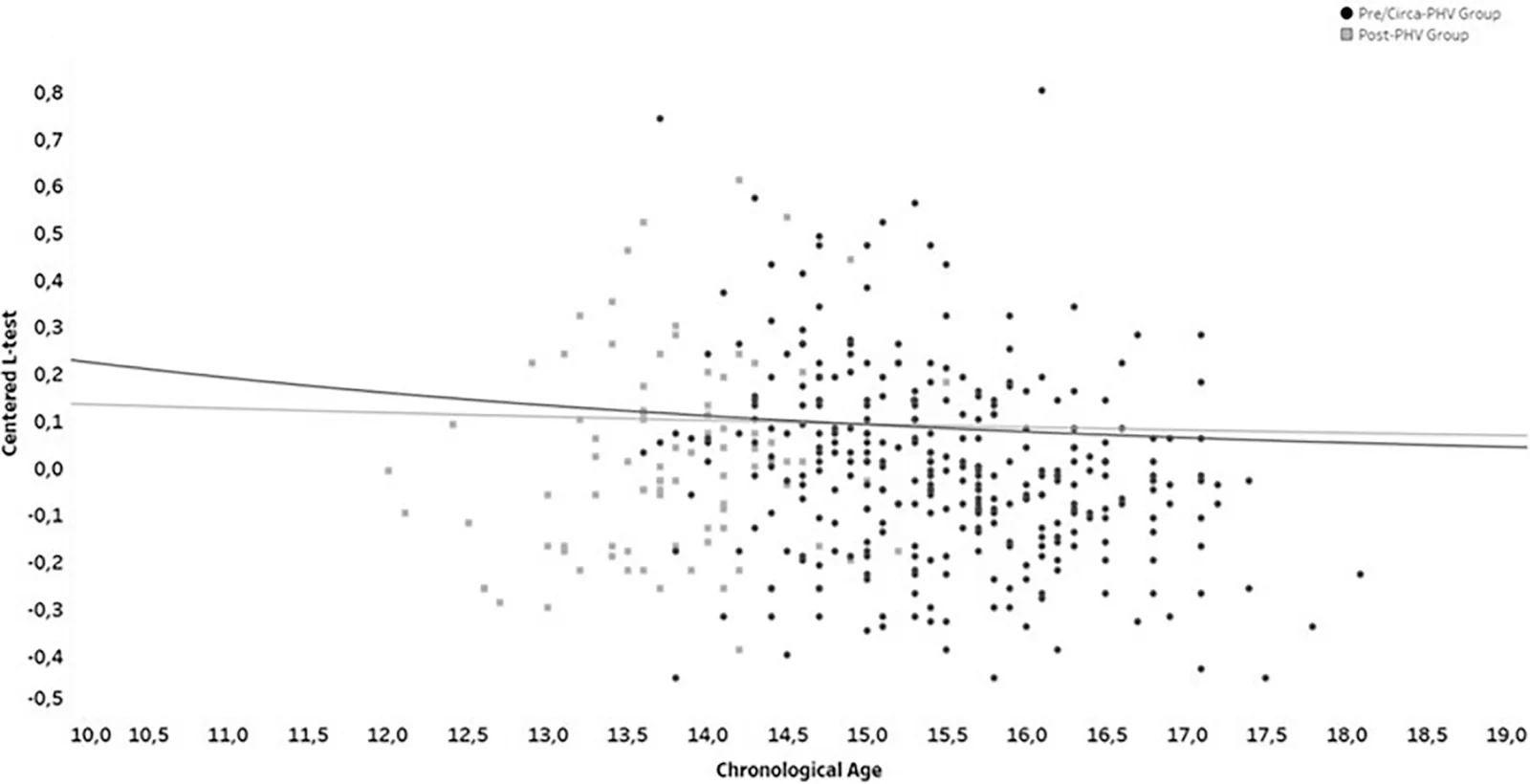
Relationship between chronological age and centered change of direction ability (L-test) according to biological maturity status (pre/circa-PHV vs post-PHV). **Key.** L-test = change of direction ability test; PHV = peak height velocity; pre-PHV = pre-peak height velocity; circa-PHV = circapeak height velocity; post-PHV = post-peak height velocity.

**FIG. 4:**
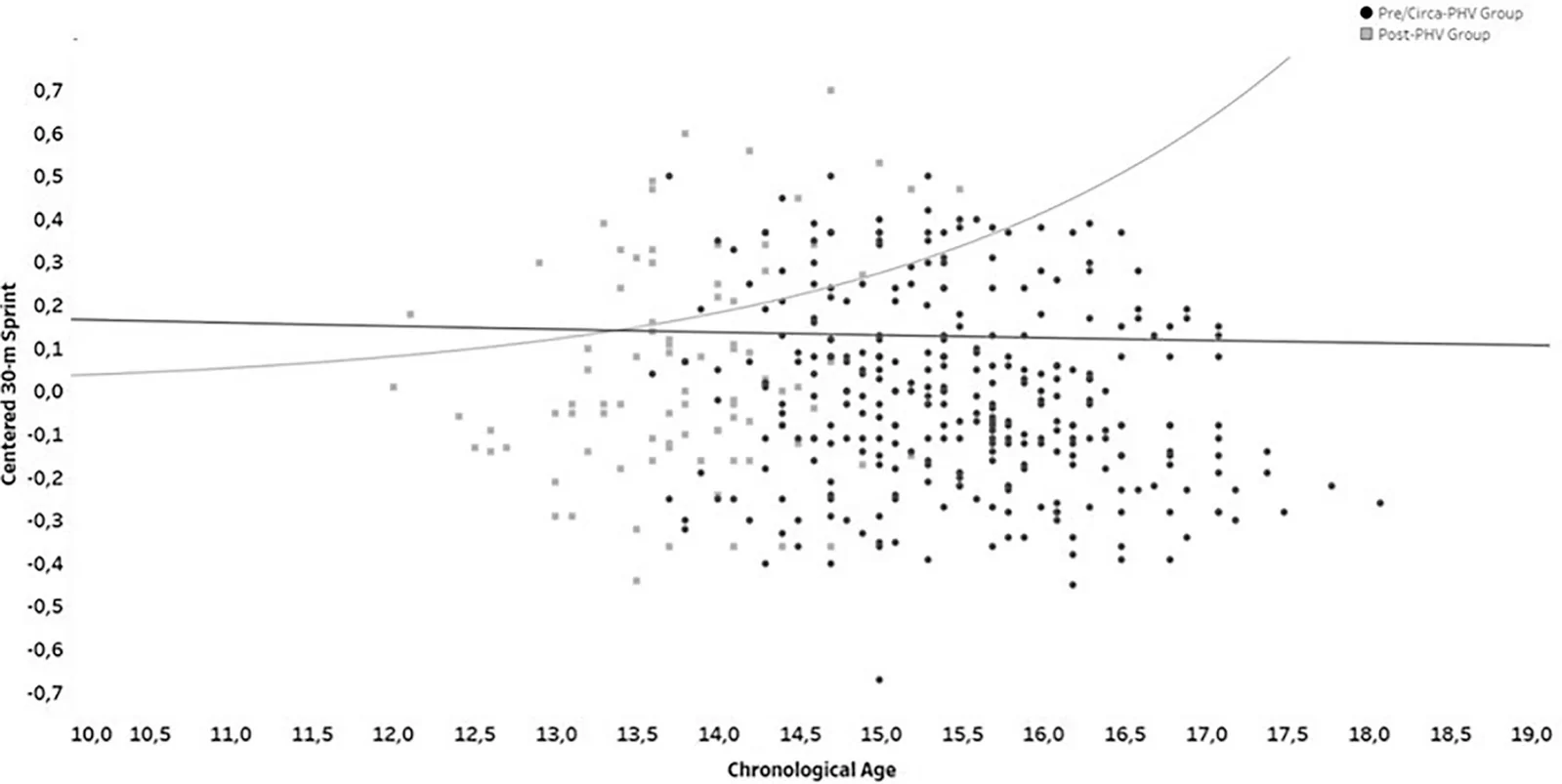
Relationship between chronological age and centered 30-m sprint performance according to biological maturity status (pre/circa-PHV vs post-PHV). **Key.** 30-m sprint = 30-meter sprint test; PHV = peak height velocity; pre-PHV = pre-peak height velocity; circa-PHV = circa-peak height velocity; post-PHV = post-peak height velocity.

**FIG. 5:**
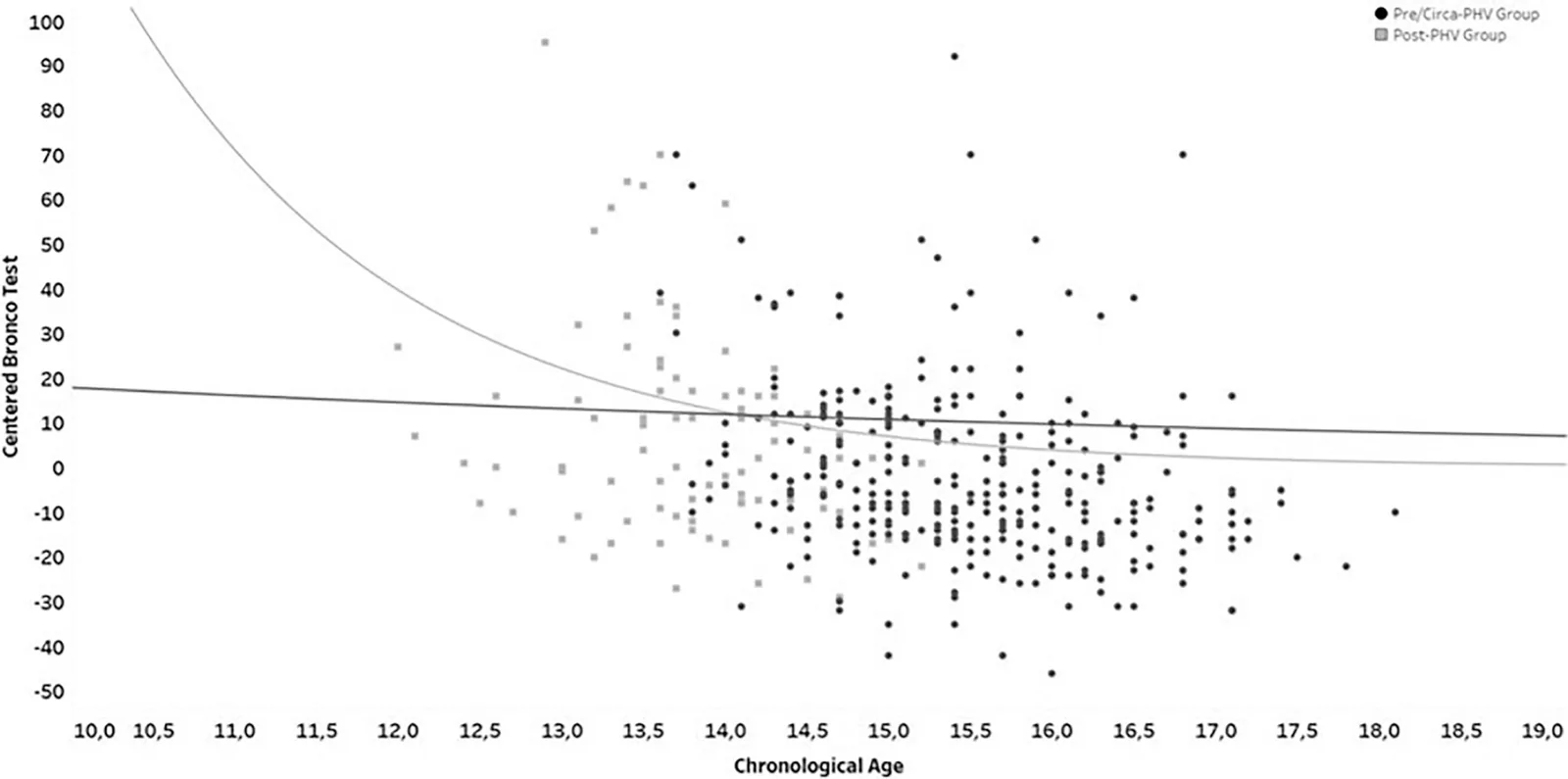
Relationship between chronological age and centered Bronco test performance (maximal aerobic speed) according to biological maturity status (pre/circa-PHV vs post-PHV). **Key.** Bronco test = maximal aerobic speed test; PHV = peak height velocity; pre-PHV = pre-peak height velocity; circa-PHV = circapeak height velocity; post-PHV = post-peak height velocity.

Model comparisons indicated that age alone provided the best fit for the L-test and 30-m sprint models. In contrast, the best-fitting models for the CMJ, Bronco, and IMTP included the interaction between age and maturity status, highlighting the importance of maturation in explaining performance changes in these tests. Detailed mixed-effects model results, including the coefficients, log-likelihood values, and fit statistics, are presented in Tables 3–5.

**TABLE 3:** Mixed-Effects Model Results for All Metrics

Test	Model	Intercept (p-value)	Age (p-value)	Maturity (p-value)	Interaction (p-value)	AIC	Log-Likelihood	Random Effect Variance
L-Test	1	0.773 (0.001)	-0.511 (0.001)	-	-	-368.437	-372.437	0.025
	2	-0.870 (0.001)	-0.055 (0.001)	-0.019 (0.420)	-	-363.520	-367.520	0.025
	3	1.332 (0.007)	-0.088 (0.009)	-0.415 (0.282)	0.028 (0.305)	-359.199	-363.199	0.024

30-m Sprint	1	0.610 (0.001)	-0.040 (0.001)	-	-	-499.238	-502.238	0.040
	2	0.623 (0.001)	-0.041 (0.001)	-0.002 (0.891)	-	-493.222	-497.252	0.040
	3	0.869 (0.029)	-0.581 (0.033)	-0.212 (0.495)	0.0148 (0.500)	-487.913	-491.913	0.040

CMJ Test	1	-42.139 (0.001)	2.786 (0.001)	-	-	2175.834	2171.834	17.918
	2	-29.533 (0.001)	2.166 (0.001)	-2.636 (0.001)	-	2154.276	2150.276	18.055
	3	12.024 (0.277)	-0.738 (0.329)	-38.529 (0.001)	2.542 (0.001)	2136.997	2132.997	19.430

Bronco Test	1	163.674 (0.001)	-10.823 (0.001)	-	-	3364.837	3360.837	239.082
	2	141.510 (0.001)	-9.742 (0.001)	4.755 (0.072)	-	3357.860	3353.860	248.691
	3	-62.001 (0.223)	4.472 (0.199)	180.659 (0.001)	-12.448 (0.001)	3335.472	3331.472	272.518

IMTP Test	1	-78.024 (0.001)	5.159 (0.001)	-	-	2253.672	2249.672	18.708
	2	-73.683 (0.001)	4.949 (0.001)	-0.954 (0.142)	-	2250.575	2246.575	19.377
	3	-46.431 (0.001)	3.045 (0.001)	-24.403 (0.016)	1.660 (0.020)	2244.043	2240.043	19.741

* (1) age as the only fixed effect; (2) age and maturity status as fixed effects; and (3) age, maturity status, and their interaction as fixed effects.

Key: L-Test = Change of Direction Ability; 30-m Sprint = Sprint Test – 30 meters; CMJ Test = Countermovement Jump; Bronco Test = Maximal Aerobic Speed (${\dot{\text{V}}\text{O}}_{2\max}$); IMTP Test = Isometric Mid-Thigh Pull.

**TABLE 4:** Results of the L-Test, 30-m Sprint Test; CMJ Test, Bronco Test, and IMTP Test.

Assessment	N	L-Test (s)	30-m Sprint Test (s)	CMJ Test (cm)	Bronco Test (s)	IMTP Test (N/kg)
1	67	6.08 ± 0.23	4.61 ± 0.25	34.9 ± 5.8	310.2 ± 27.4	23.1 ± 4.3
2	67	5.97 ± 0.20	4.47 ± 0.22	36.5 ± 5.5	292.8 ± 18.2	25.4 ± 4.9
3	67	5.96 ± 0.23	4.67 ± 0.22	37.4 ± 5.4	293.3 ± 19.7	28.3 ± 4.8
4	67	5.96 ± 0.17	4.48 ± 0.21	38.3 ± 4.6	289.9 ± 15.8	30.2 ± 5.3
5	67	5.93 ± 0.18	4.49 ± 0.22	39.6 ± 3.7	287.9 ± 16.5	30.8 ± 5.1
6	67	5.97 ± 0.17	4.50 ± 0.20	40.0 ± 4.3	284.4 ± 14.4	33.0 ± 6.0

Key: L-Test = Change of Direction Ability; 30-m Sprint = Sprint Test – 30 meters; CMJ Test = Countermovement Jump; Bronco Test = Maximal Aerobic Speed (${\dot{\text{V}}\text{O}}_{2\max}$); IMTP Test = Isometric Mid-Thigh Pull.

**TABLE 5:** Results of the L-Test, 30-m Sprint Test, CMJ Test, Bronco Test, and IMTP Test Group (Pre-PHV/PHV) and Group (Post-PHV) throughout the six assessments (two sports seasons).

Assessment	Maturity Group	L-Test (s)	30-m Sprint Test (s)	CMJ Test (cm)	Bronco Test (s)	IMTP Test (N/kg)
1	Pre/Circa-PHV	6.04 ± 0.20	4.60 ± 0.24	32.35 ± 5.11	315.24 ± 27.44	21.40 ± 4.36
	Post-PHV	6.10 ± 0.25	4.60 ± 0.25	37.13 ± 5.56	395.51 ± 27.07	24.63 ± 3.68

2	Pre/Circa-PHV	5.97 ± 0.17	4.54 ± 0.24	33.24 ± 5.63	296.88 ± 17.52	22.80 ± 4.19
	Post-PHV	5.96 ± 0.20	4.44 ± 0.19	38.10 ± 4.84	290.76 ± 18.40	26.61 ± 4.78

3	Pre/Circa-PHV	5.95 ± 0.25	4.50 ± 0.23	36.12 ± 4.25	291.53 ± 15.16	25.98 ± 4.75
	Post-PHV	5.96 ± 0.21	4.45 ± 0.22	37.77 ± 5.75	293.75 ± 20.94	28.93 ± 4.70

4	Pre/Circa-PHV	6.01 ± 0.23	4.50 ± 0.21	38.09 ± 3.72	290.76 ± 10.84	29.09 ± 4.46
	Post-PHV	5.94 ± 0.16	4.47 ± 0.20	38.34 ± 4.84	289.78 ± 16.55	30.31 ± 5.45

5	Pre/Circa-PHV	5.90 ± 0.18	4.53 ± 0.31	38.88 ± 3.42	284.36 ± 9.67	31.33 ± 7.98
	Post-PHV	5.93 ± 0.17	4.48 ± 0.20	39.62 ± 3.77	288.19 ± 17.12	30.73 ± 4.91

6	Pre/Circa-PHV	5.92 ± 0.20	4.53 ± 0.31	38.11 ± 4.05	278.83 ± 9.66	24.68 ± 7.47
	Post-PHV	5.97 ± 0.16	4.49 ± 0.19	40.22 ± 4.32	284.90 ± 14.82	32.80 ± 5.91

Key: L-Test = Change of Direction Ability; 30-m Sprint = Sprint Test – 30 meters; CMJ Test = Countermovement Jump; Bronco Test = Maximal Aerobic Speed (${\dot{\text{V}}\text{O}}_{2\max}$); IMTP Test = Isometric Mid-Thigh Pull; Pre-PHV = Pre-Peak height velocity; Circa-PHV = Circa-Peak height velocity; Post-PHV = Post-Peak height velocity;

## DISCUSSION

This longitudinal study examined the influence of biological maturation on key physical performance variables, including CMJ height, maximal strength, COD ability, sprint speed, and endurance in elite youth soccer players across two competitive seasons. The main findings were as follows: 1) biological maturation proved crucial for jumping height performance, with post-PHV players showing significant improvements over time; 2) maximal strength [IMTP test] was directly influenced by biological maturation, showing notable differences among groups, particularly in post-PHV players; 3) linear and multidirectional speed showed chronological age as the primary predictor, with older players demonstrating superior performance in specific tests (L-test and 30-m sprint); and 4) endurance [1200 m shuttle run test], where biological maturation had a marked impact, with post-PHV players showing substantial gains over time. The observed findings can be partially attributed to physiological and morphological transformations that occur during adolescence. The period circa-PHV is marked by rapid increases in muscle mass, neuromuscular coordination, and concentrations of anabolic hormones, particularly testosterone and growth hormone. These changes enhance the capacity for force and power production, which directly influence performance in tasks such as jumping and maximal strength assessments. Consequently, individuals who have surpassed PHV tend to exhibit accelerated improvements in neuromuscular performance compared to their less mature counterparts [3, 6, 41, 42].

Our analysis revealed a significant interaction between these factors (p = 0.001), indicating that the maturity level modulated the impact of age on power. Post-PHV players showed significantly greater improvements in jumping height than their pre- and circa-PHV counterparts, highlighting enhanced power development during the post-PHV period. Recent studies have investigated the relationship between biological maturation and jumping ability. A strong correlation (r = 0.63) between CMJ performance and pubertal development scale (PDS) scores has been reported in young soccer players aged approximately 13 years [43]. Similarly, a significant association between maturation, assessed using PHV, and CMJ performance in soccer players aged 8–12 years has been reported [44]. In addition, the relationships between CMJ performance, soccer category, chronological age, relative age, and pubertal development have also been investigated [45]. These findings indicate that both chronological age and pubertal development significantly influence CMJ performance, highlighting the importance of considering anthropometric and maturational variables when evaluating jumping ability in young players. Although quick movements may not be significantly affected by body size in adults, power is influenced by biological maturation, which is linked to an increase in sex hormones during puberty [44]. These studies used different methodologies to assess biological maturation (e.g., maturity offset and pubertal development scale), whereas the present investigation employed the Khamis– Roche method [21, 46].

Strength development in boys during puberty increases owing to neuromuscular adaptations and muscle morphological changes [42]. The IMTP test results underscore the significance of age and maturation on strength development. The interaction between these factors was significant (p = 0.020), demonstrating that post-PHV players achieved greater strength gains than their pre-PHV peers, demonstrating maturation’s pivotal role beyond chronological age effects. This reinforces the notion that biological maturation influences the rate at which young athletes develop maximal strength, which is consistent with previous research [41]. Our findings align with previous investigations showing that maturation impacts peak force in elite young male soccer players, with more mature players achieving superior results [47, 48]. The IMTP serves as a valuable tool for practitioners to assess players’ absolute strength and track changes during different maturation stages. Understanding these dynamics aids in creating targeted strength training regimens that optimize performance while reducing injury risk, as athletes experience varying responses based on maturation status.

In team sports such as soccer, a rapid COD is crucial for physical fitness [49], particularly COD speed [L-test]. COD speed can differentiate between top- and second-tier soccer players, making it valuable for identifying talented athletes [49, 50]. Before puberty, the COD of young men showed a steady growth. After puberty, they experience greater improvements, with peak gains around ages 13–14, aligning with PHV [51]. This indicates a two-phase maturation process. Initially, COD gains were due to neural development and enhanced motor control through improved muscle coordination [52]. During puberty, hormonal changes, including changes in growth hormones, testosterone, and IGF-1, trigger structural changes. These include muscle development, fiber type differentiation, and muscle structure modifications, which contribute to improved strength in adolescent males [50, 52]. Our COD speed analysis showed that chronological age was the main determinant of performance, with older players outperforming younger players (P < 0.001). Maturation status and its interaction with age did not significantly improve the predictive model (p = 0.420 and p = 0.305, respectively), suggesting that age alone predominantly determines COD speed in young soccer players. Previous research has reported no notable differences in COD performance between players pre- and post-PHV [53]. However, early-maturing soccer players aged 13–15 years have been reported to demonstrate superior COD performance compared with their late-maturing peers, suggesting that early maturation may confer a temporary performance advantage [54]. These contradictory results underscore the need for additional studies on the effect of maturation on COD in young soccer players [55]. Two aspects of this analysis warrant attention to previous studies: the methodology for assessing biological maturation differed from that in our study, and the selection of COD performance tests varied among studies.

Age was identified as the primary predictor of 30-m sprint performance, with older players outperforming younger players (p < 0.001). Our findings suggest that maturity status and its interaction with age did not contribute significantly to the predictive model (both p > 0.05), indicating that sprint performance in young soccer players is linked to chronological age rather than biological maturation. Although maturation did not significantly improve the statistical model, physiological evidence suggests its indirect role during development. Research has highlighted the importance of top sprint velocity in youth soccer during PHV [53, 56]. The maximum sprint speed improves during PHV, suggesting that maturation influences speed enhancement [56, 57]. However, maturation has been shown to influence peak sprint speed development during PHV, highlighting its role in sprint velocity improvements during adolescence [58]. Understanding these dynamics is crucial for creating sprint training regimens that respond to the developmental phases of young athletes. Strength and conditioning coaches can use this information to focus on age-appropriate speed training, particularly during and after PHV, to optimize sprint development and enhance players’ readiness for soccer demands. Thus, standardizing methods for assessing biological maturation and maximal speed testing protocols is vital.

The Bronco test showed that age and maturity status significantly affected the results, with a substantial interplay between the variables (p = 0.001). Young soccer players who passed the PHV exhibited greater improvement in endurance over time. Growth and maturation processes lead to physiological changes that affect energy metabolism. These changes include heart enlargement, increased blood volume, higher hemoglobin levels, possible increases in type II muscle fibers, and improved anaerobic enzyme activity [59]. Maturation leads to significant changes in the absolute peak oxygen consumption and power production through anaerobic energy systems, with notable improvements during rapid growth [6, 41]. These physiological developments contribute to improved performance in endurance and high-intensity activities through natural progression. However, maturation typically results in larger gains in anaerobic energy production than in aerobic energy production. Research has extensively studied aerobic fitness development during maturation using cross-sectional and longitudinal methods [60]. Research indicates that males experience yearly ${\dot{\text{V}}\text{O}}_{2\max}$ increases of approximately 200 mL · min^−1^ from pre-puberty to age 16 due to cardiac, pulmonary, and muscular tissue growth [60]. In our investigation, players in post-PHV showed significantly greater Bronco test distances than those in the pre- and mid-PHV stages (p < 0.01). Similar improvements during PHV have been reported previously, suggesting a transition from the pre-PHV to the post-PHV stage [61]. Our findings corroborate previous studies showing that young soccer players who passed PHV exhibited enhanced endurance over time. Recent studies have underscored the importance of accounting for biological maturation when assessing performance in young soccer players. For instance, biological maturity rather than relative age has been shown to explain a considerable proportion of selection bias among female international youth soccer players [62]. Similarly, growth and maturation have been shown to interact with training exposure and injury risk in elite soccer academies [63]. Collectively, these findings highlight the importance of considering maturation status when evaluating performance development in young soccer players.

Overall, our findings suggest that biological maturation has a taskdependent effect on physical performance. Neuromuscular qualities, such as jumping ability and maximal strength, appear to be more strongly associated with maturation status, whereas sprint and COD performance seem to be more closely related to chronological age. These differences may reflect distinct physiological determinants of performance quality, highlighting the importance of considering both maturation and age when evaluating the physical development of young soccer players. From a practical standpoint, these findings underscore the significance of accounting for biological maturation when evaluating the physical performance of elite young soccer players. Practitioners operating within academy environments should recognize that enhancements in strength, power, and endurance may be indicative of maturational processes rather than exclusively the outcomes of training. Therefore, monitoring maturation status could enable practitioners to more effectively tailor training programs, interpret performance testing results with greater accuracy, and make more informed decisions regarding talent development.

This study has several limitations that should be acknowledged. First, the relatively small number of pre-PHV players in the sample required the merging of pre-PHV and circa-PHV players into a single group, which may have reduced the sensitivity to detect potential differences between the early stages of biological maturation. Second, biological maturation was estimated using the PPAH derived from the Khamis–Roche method rather than direct measures such as skeletal age assessment. Although this method is widely used in youth sports research, it can introduce errors in the estimation. Therefore, the potential misclassification of maturity status due to this estimation error should be considered when interpreting the results. Third, the sample consisted of players from a single elite soccer academy, which may limit the generalizability of the findings to other developmental environments. Therefore, future studies should include players from multiple academies and consider additional contextual factors, such as training load and soccer-specific exposure, when examining the relationship between biological maturation and physical performance.

### Practical Applications

These findings provide actionable insights for sports scientists, coaches, and young soccer practitioners seeking to optimize the training and development of young soccer players. By considering chronological age and biological maturation, professionals can more effectively individualize training programs to align them with each player’s developmental profile. For instance, power, strength, and endurance training could be intensified in post-PHV players, who demonstrated more substantial improvements in physical qualities than their pre- and circa-PHV counterparts. Such tailored approaches can maximize gains in power, endurance, and maximal strength, ensuring that training efforts are congruent with each athlete’s maturation status.

The integration of biological maturation assessments can also enhance talent identification processes. By accurately assessing maturation status, programs can mitigate the risk of misidentifying latematuring players, ensuring that talent potential is evaluated more comprehensively than relying solely on chronological age. Furthermore, practitioners can utilize the study results to design long-term athlete development plans and adjust training loads and competition demands according to each player’s growth stage, which may reduce injury risk and support sustained performance improvements over time.

This study further supports the development of a comprehensive battery of longitudinal tests to track performance in young soccer programs. This data-driven approach enables academies to monitor players’ progress, compare results across teams or academies, and establish benchmarks to inform individualized training strategies. The analytical models derived from this study’s findings can also aid in predicting performance outcomes, allowing soccer academies to make informed, evidence-based decisions in player development. Integrating maturation levels into talent identification and development processes is essential for accurately evaluating young athletes and mitigating the potential biases linked to developmental timing.

## CONCLUSIONS

Chronological age was the primary predictor of COD and sprint performance in elite young soccer players, with older players generally demonstrating superior results in these tasks. In contrast, biological maturation has a stronger influence on neuromuscular and endurancerelated qualities. Specifically, maturation status was associated with improvements in CMJ performance, maximal strength (IMTP), and MAS, with post-PHV players exhibiting greater performance gains over time. These findings highlight the importance of considering both chronological age and biological maturation when interpreting the development of physical performance in young soccer players.

## Data Availability

The datasets supporting the conclusions of this article are available from the corresponding author upon reasonable request.
